# Probe-based confocal laser endomicroscopy for *in vivo* evaluation of the tumor vasculature in gastric and rectal carcinomas

**DOI:** 10.1038/s41598-017-10963-1

**Published:** 2017-08-29

**Authors:** Paola Spessotto, Mara Fornasarig, Eliana Pivetta, Stefania Maiero, Raffaella Magris, Maurizio Mongiat, Vincenzo Canzonieri, Paolo De Paoli, Antonino De Paoli, Angela Buonadonna, Diego Serraino, Chiara Panato, Claudio Belluco, Renato Cannizzaro

**Affiliations:** 1 Molecular Oncology, Department of Translational Research, CRO-IRCCS, National Cancer Institute, Aviano, Italy; 2 Gastroenterology, CRO-IRCCS, National Cancer Institute, Aviano, Italy; 3 Pathology, CRO-IRCCS, National Cancer Institute, Aviano, Italy; 4 Scientific Directorate, CRO-IRCCS, National Cancer Institute, Aviano, Italy; 5 Radiation Oncology, CRO-IRCCS, National Cancer Institute, Aviano, Italy; 6 Medical Oncology, CRO-IRCCS, National Cancer Institute, Aviano, Italy; 7 Epidemiology and Biostatistics, CRO-IRCCS, National Cancer Institute, Aviano, Italy; 8 Surgical Oncology, CRO-IRCCS, National Cancer Institute, Aviano, Italy

## Abstract

Probe-based Confocal Laser Endomicroscopy (pCLE) is a powerful imaging technique that allows to perform gastrointestinal endomicroscopy at subcellular resolution. The aim of this study was to assess the use of pCLE to evaluate tumor angiogenesis in rectal and gastric cancers. A total of 35 consecutive patients with gastric and 91 with rectal carcinomas underwent endoscopy and pCLE during the same examination. Vascular assessment was based on vessel shape and size, vessel permeability and blood flow, and allowed the creation of an angiogenic score ranging from 0, for normal vasculature, to 4, for aberrant vasculature. A significant difference for the presence of vessels with large diameter and defective blood flow was found between rectal and gastric cancers. Overall, rectal cancers displayed a higher angiogenic score compared to gastric cancers. Conventional therapy induced a striking reduction in the angiogenic score only in rectal cancer patients. Taken together, our findings suggest that the pCLE technology is suitable for the evaluation of the tumor microvasculature abnormalities. Therefore, the real-time assessment of the vasculature status may represent a promising approach to predict the efficacy of the treatments and improve the clinical management of patients with gastric or rectal carcinomas.

## Introduction

Gastrointestinal (GI) cancer represents a major cause of morbidity and mortality, with incomplete response to chemotherapy in the advanced stages and poor prognosis^1, 2^.

The angiogenic switch is a crucial step in tumor progression where the balance between pro- and anti-angiogenic factors tilts toward the formation of new vessels to supply tumors with oxygen and nutrients for their relentless growth. The aberrant angiogenic stimuli within tumors leads to the formation of an abnormal vascular network characterized by dilated, tortuous, and hyper permeable vessels^3–5^ that diminish the efficacy of therapeutic treatments. Thus, evaluation of the tumor vascular pattern can provide vital information with a view to achieving a more accurate diagnosis and patient-tailored targeted treatment^5^. Recently, several studies have highlighted the importance of assessing the vascular pattern of gastric and rectal cancers in order to improve the accuracy determining the prognosis and establish the appropriate treatment^6–9^. Currently, immunohistochemistry is a commonly used method in preclinical and clinical studies for assessing tumor vascularization and determining microvessel density. This method is time-consuming and involves invasive biopsy sampling to monitor vascular changes over the treatment period^10^. Besides vascular density, other morphologic and functional parameters such as vessel diameter and shape, branching points and vessel permeability can be taken into account with tumor angiogenesis^11–13^.

Probe-based Confocal Laser Endomicroscopy (pCLE) is an advanced endoscopic technique that can provide a dynamic real-time imaging of the mucosa at subcellular level^14, 15^. The visualization of the cellular and subcellular structures, as well as capillaries and single red blood cells, are peculiar characteristics of this novel GI endoscopy method. High-resolution confocal imaging is achieved through i.v. injection of fluorescein^16^. This enables analysis not only of the vascular structure but also of the functionality, since the dye pours out of the abnormally formed vessels and the extent of the leakage can be determined. In fact, in a recent study, vessel permeability was evaluated with this approach in patients with ulcerative colitis^17^. This technique has also been used in a few studies to objectively evaluate the microvessel density in different neoplastic stages. Preliminary data of the microvessel density have been reported for biliary cancers at the liver hilum^18, 19^, for Barrett’s esophagus^20^, and for GI tumors^21–23^.

Based on this preliminary evidence, the intention of this study was to exploit this new promising imaging tool to analyze the angiogenic pattern in patients with gastric and rectal cancer. The aim was to provide, by means of a non-invasive technique, a prompt and accurate evaluation of the pattern and efficiency of intratumoral vessels which may be important for predicting the efficacy of chemotherapy.

## Results

### Definition of the “angiogenic score”

**Figure 1:**
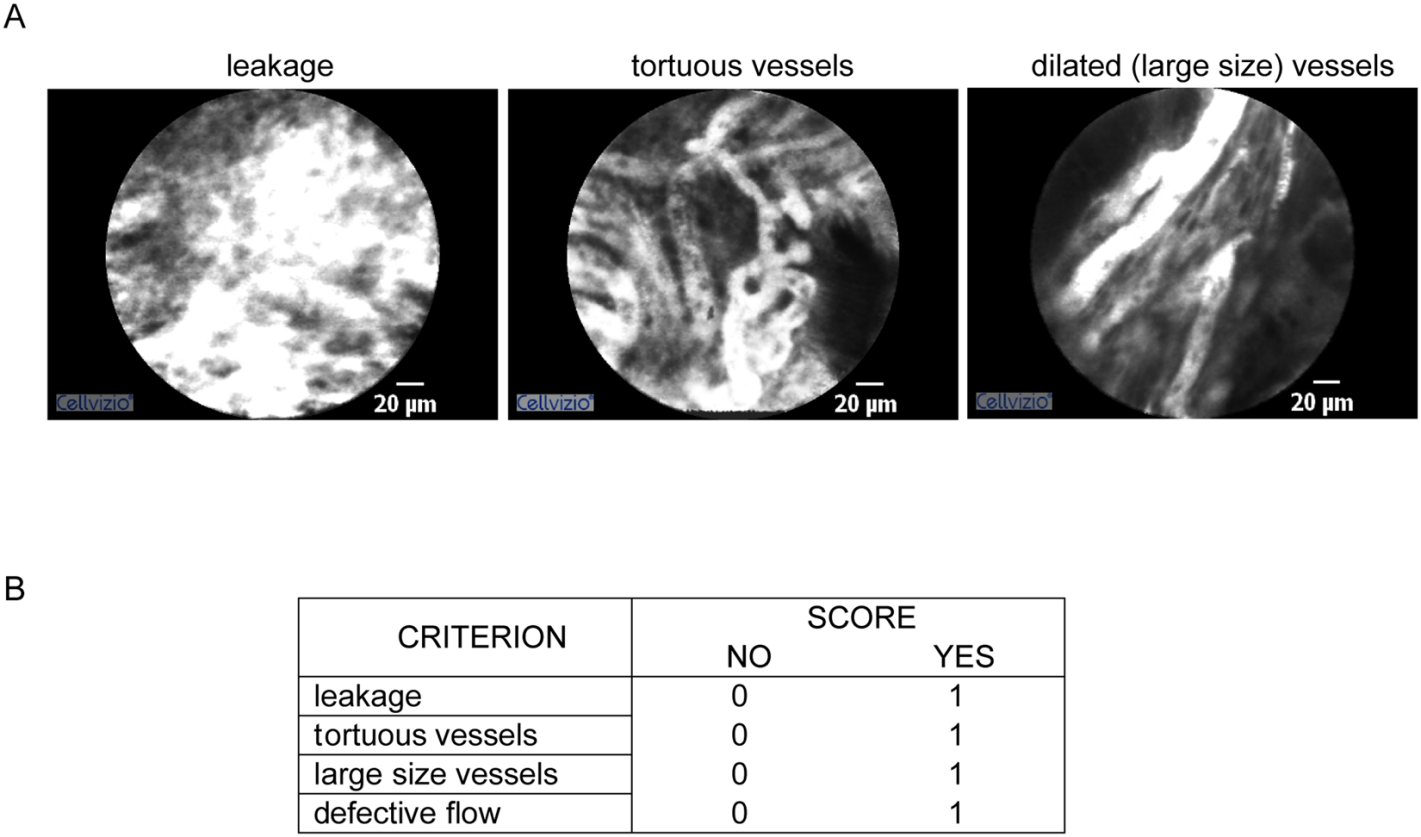
Angiogenic score parameters. (**A**) Representative images obtained during pCLE endomicroscopy showing the presence of vessels’ leakage, tortuous vessels, and dilated, large blood vessels. (**B**) Table reporting the criteria used to generate the angiogenic score, i.e. through the arithmetical sum of the scores of the single parameters to which a value of 0 was assigned to indicate absence and of 1 to indicate presence.

In this investigation we specifically focused the attention toward the vasculature pattern at the surface of the tumors. In all patients the blood vessels were clearly visible and well resolved by pCLE and it was possible to discriminate the typical aberrant features characterizing the tumor-associated blood vessels. The vascular architecture of all the vessels was abnormal, being mostly represented by enlarged, distorted and highly permeable microvessels with altered erythrocyte flow. The representative images reported in Fig. 1A clearly indicate the presence of dilated, large and tortuous vessels, as well as of leakage of fluorescein from the vessels into the highly vascularized tumoral tissue. These vessels are often characterized by a discountinuous and defective blood flow (see Video S1). A value of “1” was assigned for the presence of each parameter and a value of “0” for the absence. Based on these observations, we generated an “angiogenic score” ranging from 0 (normal vasculature) to 4 (extremely aberrant and non-functional vasculature) and representing the arithmetical sum of the single parameters as reported in Fig. 1B.

**Figure 2:**
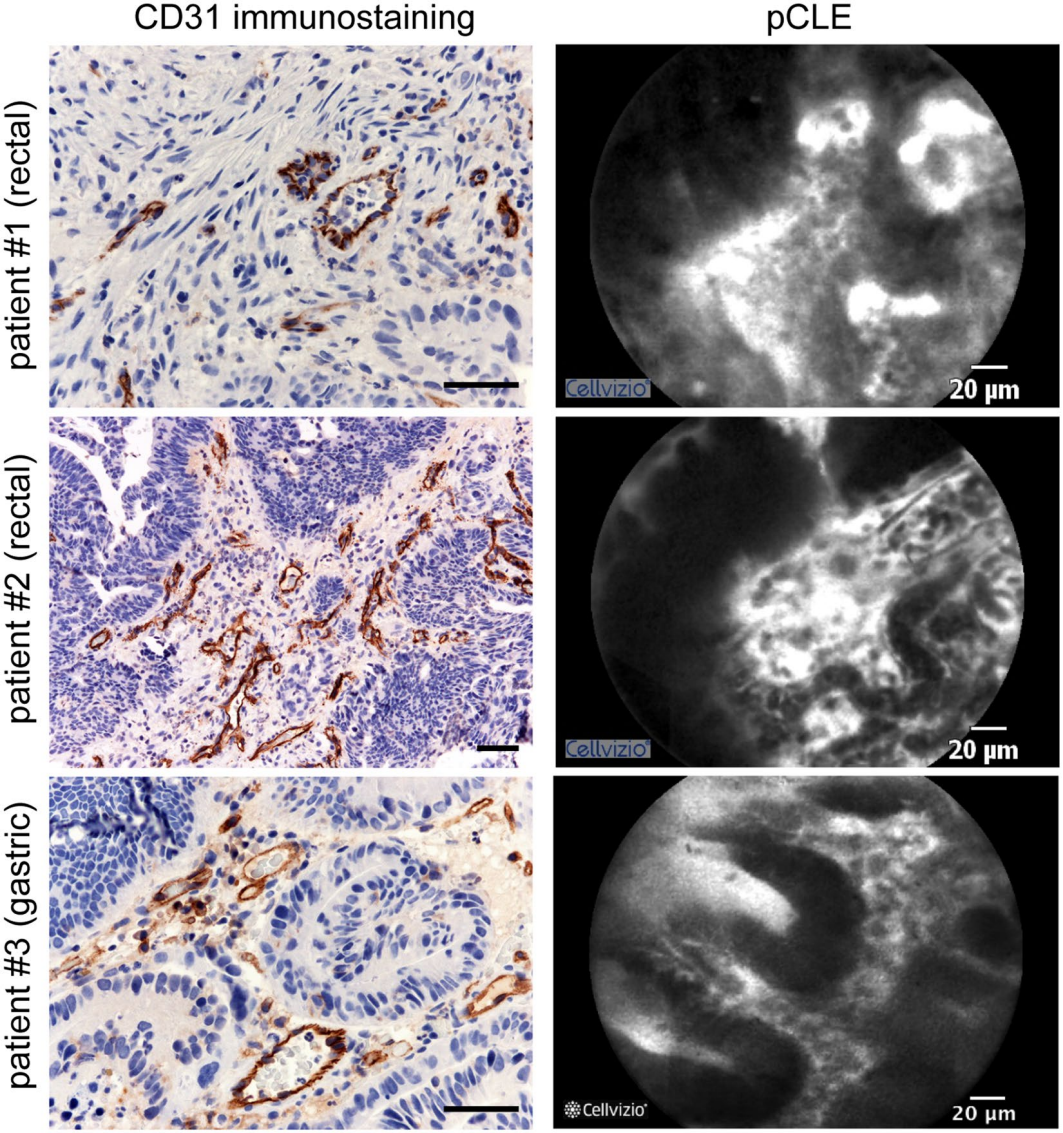
CD31 immunohistological staining and pCLE imaging. Left panels: Representative images of the histological analysis of the tumor sections from three patients (rectal, patient #1 and #2; gastric, patient #3) using the anti-CD31 antibody to detect the blood vessels. Scale bar: 50 µm. Right panels: Representative pictures of the images obtained during the pCLE endoscopic analysis from the same patients.

To assess the reliability of the angiogenic score we then compared vessel patterns identified with the pCLE analyses with the immunohistochemical analyses of the colon biopsies following CD31 staining. In a first set including 40 consecutive patients, pCLE images from 25 sequences in the same area of the biopsy sites were qualitatively compared to the CD31-stained vessels from the corresponding histological section. In Fig. 2, the images from the representative sequences obtained from three patients (#1 and #2, rectal; #3, gastric) by pCLE are compared side by side with the corresponding CD31 immunohistological staining. The comparison showed a qualitative clear overlap between the results obtained through the immunohistological analysis and the morphological patterns assessed in real time by pCLE. Encouraged by these results the remaining patients with advanced-phase cancers were subjected to pCLE analysis.

### Angiogenic score in rectal and gastric cancers

**Figure 3:**
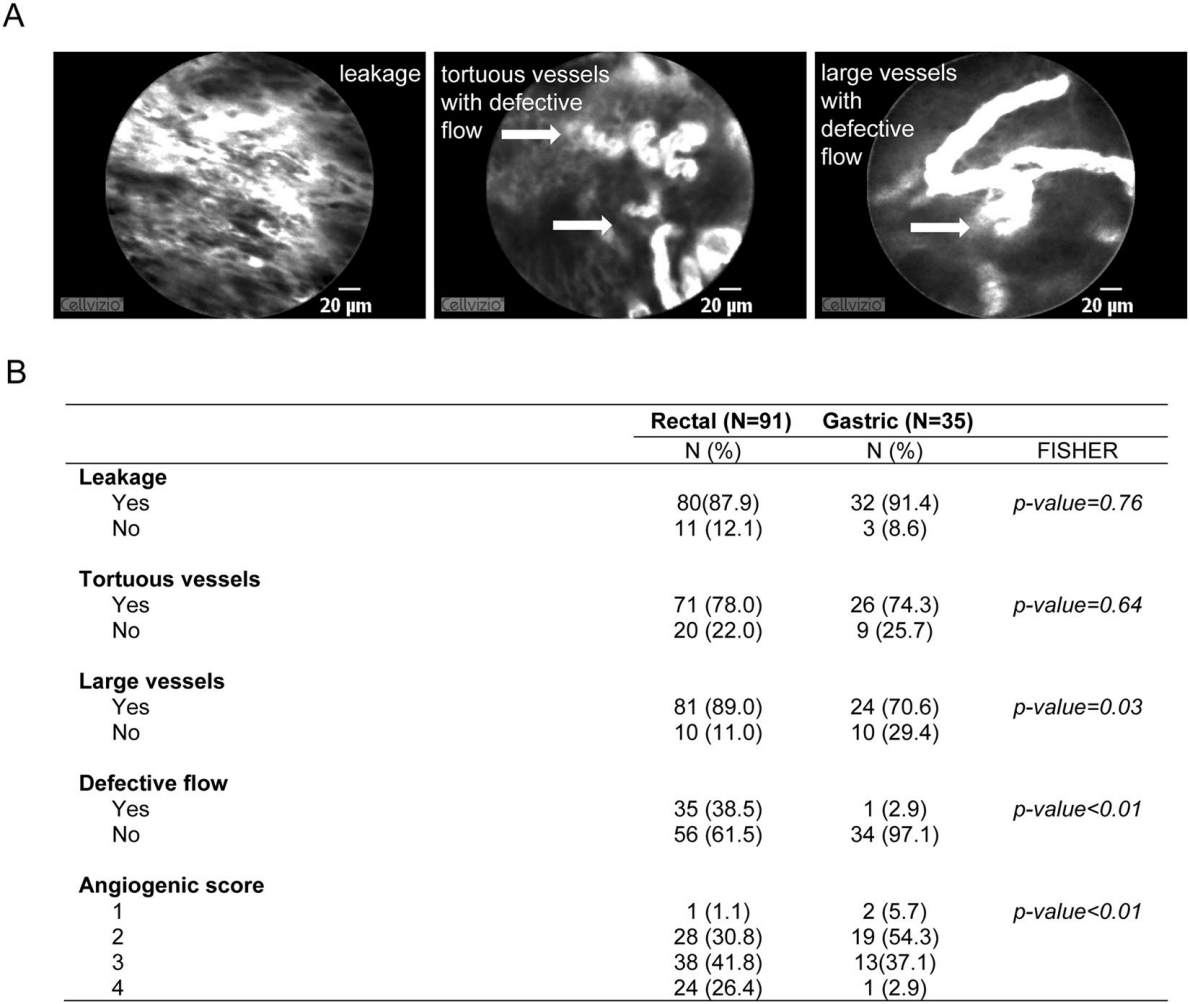
Angiogenic score in rectal and gastric cancers. (**A**) Representative images obtained from a rectal cancer patient displaying a high angiogenic score (4). In this patient all the altered features of tumor vasculature taken into account (leakage, tortuous and large vessels, and aberrant flow) were present. The white arrows indicate typical areas of discontinuous blood flow. (**B**) Table reporting the percentage of gastric and rectal cancer patients displaying the morphological and functional parameters mentioned above and the distribution of the angiogenic score among all rectal and gastric cancers analyzed. The presence of large vessels and defective flow was significantly more frequent in rectal than in gastric cancers.

The angiogenic score was assigned to every patient undergoing pCLE and none of the patients scored “0”, due to the fact that the tumors were locally advanced and that vasculature never appeared “normal”. An example of highly abnormal vasculature is shown in Fig. 3A. In this case, an angiogenic score of “4” was assigned to the tumor vasculature since all angiogenic parameters taken into account to measure the vessel abnormality were present. Moreover, more than one parameter could be detected in a single frame (see Fig. 3A, right and middle images). In this rectal cancer patient, the presence of aberrant vasculature was particularly evident, especially in terms of defects in blood flow, implying inefficient blood transportation. In this clinical case we also observed an abundance of very irregular, tortuous and dilated vessels, characterized by uneven orientation with the adjacent tissue and by frequent regions of leakage (see Fig. 3A).

Taking into account both gastric and rectal cancer patients, the first observation was that the vasculature differed qualitatively in the two types of tumors, especially regarding the presence of aberrant blood flow. This was rarely detectable in gastric cancer, whereas it repeatedly occurred in the vessels of the rectal cancer in a statistically significant way (P < 0.01, see Fig. 3B). Also vessels with a large diameter were more frequent in rectal than in gastric cancer patients (P = 0.03, see Fig. 3B). The other two abnormalities taken into account were homogeneously distributed in both cancers (leakage, P = 0.76; tortuous vessels, P = 0.64).

Gastric and rectal cancers were found to be highly angiogenic since the score “1” was assigned to few cases in both tumor types (6% and 1%, respectively; see Fig. 3B). The percentage of the scores varied between the two tumors (P < 0.01). A striking difference could be observed for the highest score documented in 26% of the rectal cancers and only in 3% of the gastric cancers (see Fig. 3B). Indeed, a higher angiogenic score was more frequently observed in rectal than in gastric cancer patients.

### Angiogenesis score and tumor grading

A total of 110 patients underwent both pCLE analysis and endoscopic ultrasonography. These patients (35 gastric and 75 rectal cancers) were distributed into two categories (T2/T3N0, and TxN+) according to the presence or absence of lymph node infiltration (N0, no infiltration; N + , lymph node infiltration). As shown in Fig. S1, we only observed that the majority of the rectal cancers with lymph node infiltration (79.4%) were included in categories with high (3 and 4) angiogenic scores. Despite not being statistically significant and the need to examine a larger number of patients, this finding suggested that the evaluation of the aberrant features obtained through the pCLE analyses was associated more with tumor progression in rectal than in gastric cancer patients, where no association between angiogenic score and tumor grading was found at all.

### pCLE in patients after radio/chemotherapy treatment

**Figure 4:**
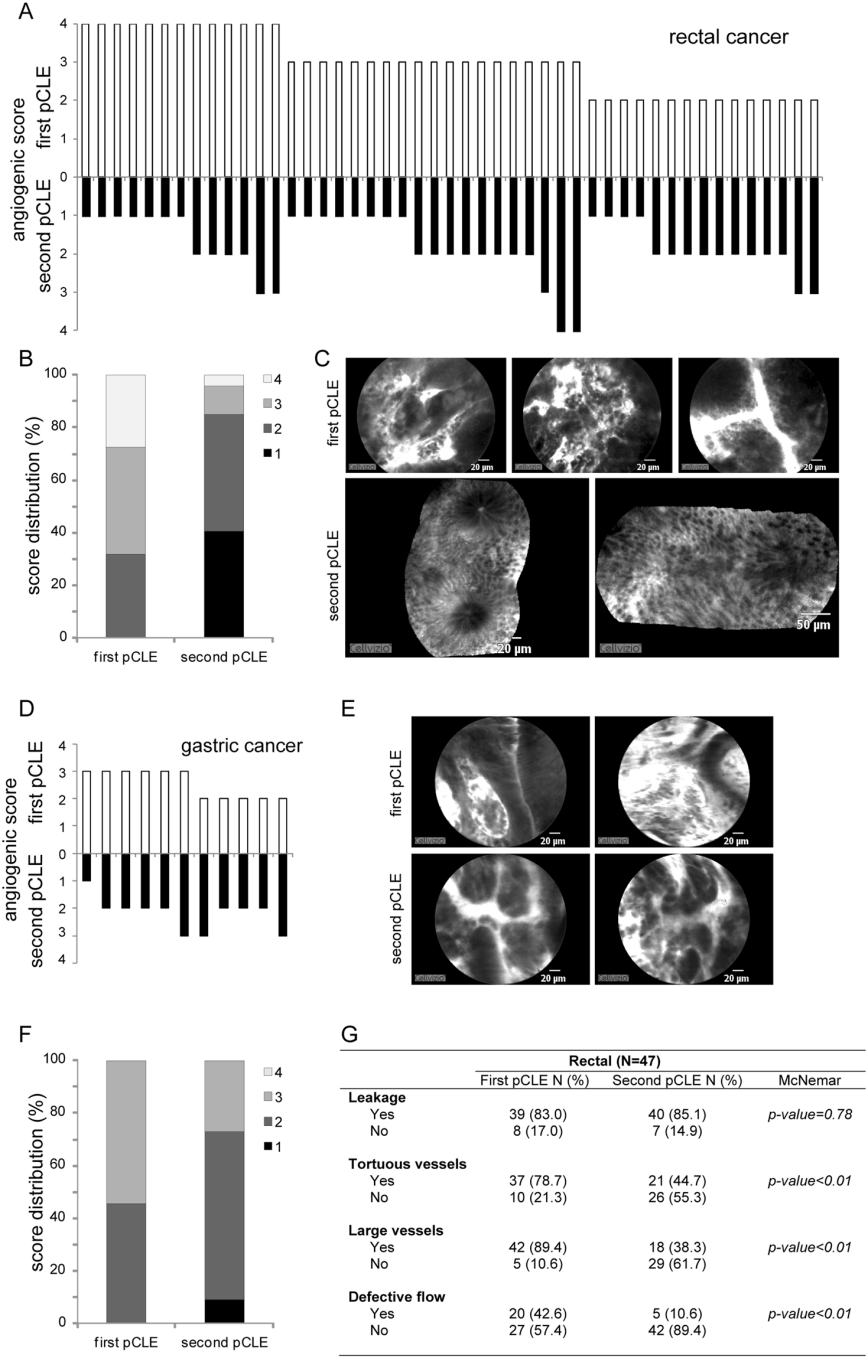
pCLE analysis after radio/chemotherapy. Graphs showing the angiogenic scores (**A** and **D**) and score distribution (**B** and **F**) assigned before (first pCLE) and after therapy (second pCLE) in 47 rectal and 11 gastric cancer patients. Representative pCLE images collected from a patient affected by rectal (**C**) or gastric (**E**) cancer before and after therapy. (**G**) Table reporting the percentage of rectal cancer patients displaying the morphological and functional parameters taken into account to generate the angiogenic score assessed before and after therapy.

A total of 58 out of 126 patients completed the program of neoadjuvant radio/chemotherapy (47 with rectal and 11 with gastric cancer) corresponding to 46% of the total number of patients (52% of total rectal and 31% of total gastric cancers) and were re-evaluated by pCLE. The evaluation of the vasculature after therapy (second pCLE) indicated an overall reduction of the angiogenic score in almost all rectal cancer patients (see Fig. 4A–C). Indeed, only 4 patients (6%) were characterized by an increased angiogenic score and 10 (21%) displayed no changes (see Fig. 4A). pCLE results were in agreement with the standard endoscopy showing that a lower angiogenic score corresponded to a clear reduction of the tumoral area, whereas in the case of clinical progression an increased angiogenic score could be detected (data not shown). Interestingly, a superior score reduction was observed when in the first pCLE (before treatment) tumors were characterized by a high angiogenic score (4 or 3) (see Fig. 4A). Furthermore, unlike the presence of tortuous and large vessels and of aberrations of the blood flow which were significantly reduced after therapy, the presence of leaky vessels did not change (see Fig. 4G). No evident changes were detected in gastric cancers after therapy (see Fig. 4D–F, and Table S2). In this case the vasculature displayed overall the same alterations detected before the treatment, indicating that the therapy did not affect angiogenesis in this type of tumor.

### On-line and off-line evaluation of angiogenesis score

**Figure 5:**
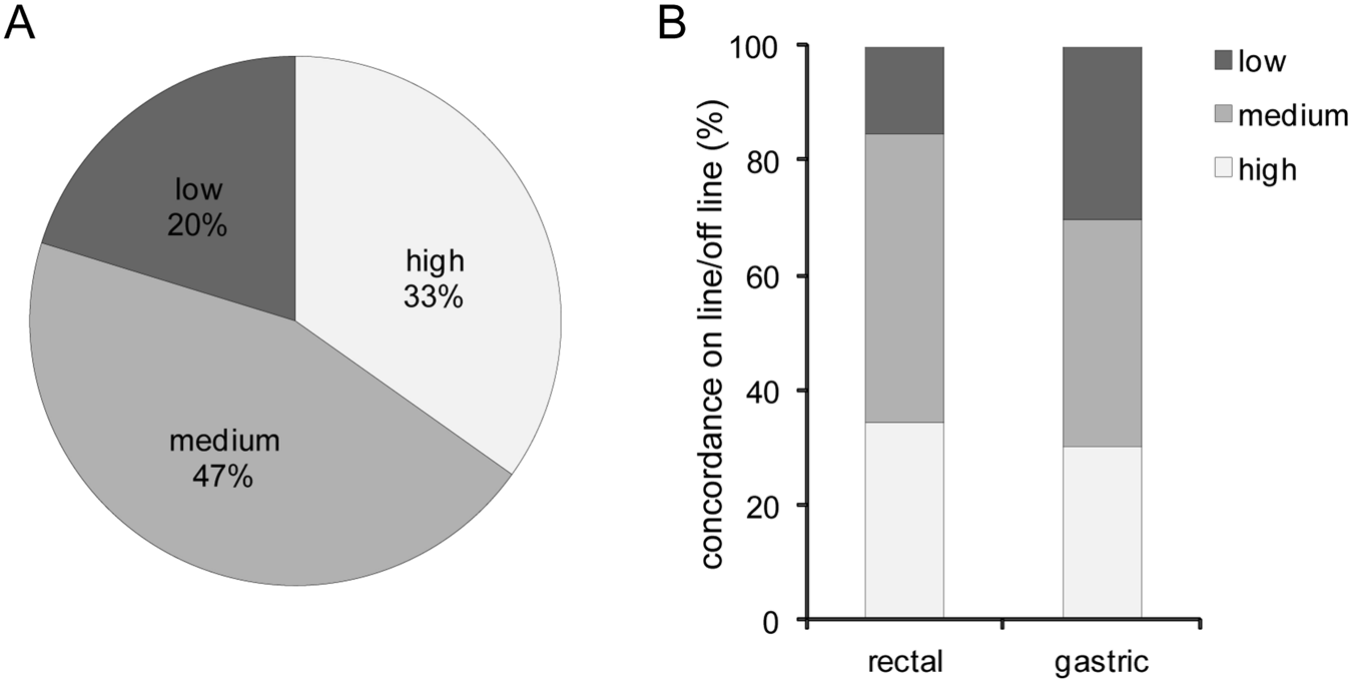
On-line and off-line evaluation of the angiogenesis score. (**A**) Graph representing the consistency between the on-line and off-line evaluations of the angiogenic score. “High” correlation was attributed to the cases where the same value was assigned during the on-line and off-line analysis section; “medium” correlation when the scores differed for “1” and “low” for “2”. (**B**) Correlation between the on-line and off-line evaluations for rectal and gastric cancers analyzed separately.

A first evaluation of angiogenic status was given in real time by the endoscopist during the pCLE analysis (on-line evaluation). Images were stored digitally and then reviewed with a dedicated software package. pCLE sequences were put into a random order and analyzed by a single investigator who was fully trained in confocal image interpretation and blinded to any clinical, endoscopical, or histopathological data (off-line evaluation). To verify the possibility of obtaining measurable information about the angiogenic status of the patients at the time of the endoscopy procedure, we then compared the given scores, considering three grades of concordance: “high” when the same score was assigned to a given patient; “medium” when the score differed by “1” and “low” when it differed by “2”. This approach was applied for all the pCLE analyses performed and for gastric and rectal cancers separately. A perfect (high) concordance was found in 33% of all endoscopies performed by pCLE whereas in 20% the concordance was low (see Fig. 5A). Although not statistically significant (P = 0.07) the percentage of low consistency obtained with the analysis of gastric cancers was higher (30%) than that obtained with the analyses of rectal cancers (16%), suggesting that the endoscopists were able to more consistently and properly evaluate the vascular network in this latter type of cancer (Fig. 5B).

## Discussion

In this study, we explored the potential employment of pCLE for assessing the extent and quality of the tumor vascularization in two pathologic conditions of the GI tract. So far, the pCLE technique has been successfully employed for the detection of neoplastic Barrett’s esophagus^24, 25^, as well as for gastric and colorectal neoplasias^26–28^. These studies have demonstrated that the use of pCLE empowers the diagnosis with predictive information leading to clinical benefits for the management of GI neoplasias. However, confocal imaging has the potential to go far beyond. The results obtained in this study suggest that this technique is exceptionally useful for visualizing tumor vasculature. As suggested by other authors, a non-invasive characterization of the tumor vessels may be useful for predicting the effects of the therapy and be of key importance for the development of personalized therapeutic interventions^29, 30^.

The use of pCLE for visualizing the angiogenic status of tumors could provide clinicians with fast and accurate information in contrast to the traditional conventional immunohistochemical approach. The present investigation is the first to take into account the analysis of tumor vasculature by pCLE in a considerable number of rectal and gastric cancers. Two recent studies showed that the use of confocal laser endomicroscopy in colorectal cancers was suitable for measuring more vascular parameters than conventional immunohistochemistry alone, and for detecting tumor neoangiogenesis with fluorescently labeled antibodies^31, 32^. The approach used by the authors was completely different: the pCLE analysis was not performed during the endoscopic examination but on fresh tumor biopsies. Moreover, in these studies only a very small number of colon cancer patients were enrolled for the analysis. Very few patients were also examined in another very recent study showing that pCLE could be a valid technique for assessing the microvascular networks in colorectal cancers during routine endoscopy^33^.

To quantify the grade of alteration of the tumor vasculature we decided to take into account the most important features characterizing the tumor-associated vasculature that could also be easily detectable with pCLE. We previously reported that the alterations of the tumor vasculature could be useful for providing information about the “angiogenic status” of the patients^34^. In the present study the analysis of a consistent number of patients allowed the generation of an “angiogenic score” based on the presence of vessel leakage, dilated (large) and tortuous vessels and the presence of defective blood flow. To detect this last feature, the pCLE approach is indispensable since only a moving picture can record the back and forth flow of blood cells. Furthermore, endomicroscopy is also unique in its ability to dynamically visualize cellular processes in their native environment free of artifacts^23, 35^. Thus, given the extended possibilities offered by this new technique, we aimed to evaluate different vascular morphometric parameters in rectal and gastric cancers, which were previously difficult to achieve through conventional immunohistochemistry. We found that, although both advanced rectal and gastric cancers were highly angiogenic, the two tumor types were significantly different concerning the frequency of dilated vessels and the presence of defective flow, which were more detectable in the rectal tumor vasculature. As a consequence, the angiogenic score was generally higher in rectal than in gastric cancer. These results may entail two distinct consequences. On the one hand, the defective flow may profoundly affect the escape of tumor cells in the blood to invade new organs and thus the progression of the disease may be affected by distinct contributions in the two tumor types. On the other side, the presence of a highly non-functional vasculature may jeopardize the therapeutic management of these tumors. It is in fact known that the delivery of chemotherapy can be highly compromised in the presence of an exceedingly abnormal vasculature^36, 37^. Only the normalization of these vessels, possibly through the employment of anti-angiogenic drugs, may make it possible to achieve more efficacious treatments for patients. Therefore, to be able to assess the extent and quality of the blood vessels within the tumors and also to monitor the possible modifications occurring during treatments, we conducted a preliminary evaluation using pCLE also after neoadjuvant radio- and/or chemotherapy. While in the majority of rectal cancer patients we detected a reduction of the angiogenic score after treatment, no substantial changes were documented in gastric cancers. The presence of leaky vessels did not change in general, possibly due to the inflammatory process activated by the therapy. The decrease of the angiogenic score in rectal cancers was indicative of an efficacious therapeutic intervention; on the other hand, the unchanged vascular alterations in gastric cancer patients correlated positively with stable or progressive disease. Indeed, since a small remission was detected in only 27% of gastric cancer patients (data not shown) it was possible that the unaltered angiogenic score could be ascribed to the lack of treatment efficacy. Based on these findings, it can be reasoned that, during the treatment schedule, pCLE analysis could be carried out to monitor the changes in the tumor vasculature induced by the treatments and possibly introduce anti-angiogenic drugs when necessary. Moreover, the analyses performed on gastric cancer patients suggest that these patients could especially benefit from neoadjuvant angiogenetic tailored therapy, thereby improving also the efficacy of the standard therapy. A greater benefit with anti-angiogenic strategies had already been established for the humanized anti-VEGF-A monoclonal antibody bevacizumab. The antibody failed to yield an OS advantage as demonstrated in the international AVAGAST trial but enabled significant improvements in progression-free survival (PFS) and response rate^38^. Two other recent trials (REGARD and RAINBOW) confirmed the survival advantage of the antiangiogenic agent ramucirumab, an antibody against VEGF-R2, used in second-line setting in gastric cancer^39^.

Our study shows that the angiogenic score may be applied during endomicroscopy with a moderate grade of “consistency”, at least for rectal cancer patients, thereby granting very rapid information on the vascularization pattern of a given patient. A lower concordance related to gastric cancer analyses could be due to the excess of fibrotic tissue in gastric tumors, which may render difficult the clear detection of the vascularized regions by pCLE in real-time. This problem is overcome by off-line evaluation since the dedicated software allows the images to be corrected and stabilized after digital storage. In any case, off-line evaluation can provide information more rapidly than histological procedures.

The evaluation of tumor vasculature may provide vital information for predicting the efficacy of treatments and for achieving tailored interventions for individual patients. Here we showed that pCLE is a valid method for real-time analysis of the vascular density and efficacy in gastric and rectal cancers. The results indicate that pCLE has the potential to generate a significant impact and there is a tangible possibility of translating the information gathered into clinical practice. By recording the morphological and “functional” characteristics of tumor vessels by assigning an angiogenic score to each patient during endoscopy, we could identify those patients who might benefit from anti-angiogenic therapy.

## Materials and Methods

### Patients

A total of 126 patients with locally advanced cancer in the majority of cases (35 had gastric cancer and 91 rectal carcinoma) who underwent pCLE endomicroscopy between January 2014 and April 2016 were consecutively enrolled. Written informed consent was obtained from each patient on the day of the procedure. The methodologies conformed to the standards set by the Declaration of Helsinki. This study was approved by the Institutional Board of CRO-IRCCS, National Cancer Institute of Aviano (PN), Italy (IRB no. CRO-2014-03). The clinical evaluations are reported in Table S1. Rectal cancer patients underwent the standard treatment for locally advanced rectal cancer (neoadjuvant radiation with concurrent fluoropyrimidin-based chemotherapy, followed by surgical resection including total mesorectal excision). Gastric cancer patients underwent neoadjuvant multiregimen chemoradiotherapy followed by surgical resection. Laboratory and pathological results were collected by means of the hospital database.

### Endoscopy procedures and pCLE analyses

pCLE analyses were carried out with GastroFlex UHD probe (Cellvizio, Mauna Kea Technology, Paris, France) during gastroscopy and colonoscopy (Olympus series 180). 110 patients also underwent endoscopic ultrasonography (Olympus series 160) immediately after pCLE analyses. Patients were examined before any radio/chemotherapeutic or surgical intervention (first pCLE). 58 patients (corresponding to 46% of all patients) were also examined after treatment (second pCLE). Images and sequences of the normal and neoplastic mucosa were taken and the conventional bioptic samples obtained by macrobiopsy (COOK Medical, Ireland) at the end of the examination. Images were recorded within the first 10 minutes following i.v. injection of fluorescein (5 ml of a 10% solution). pCLE images were collected at 12 frames per second to ensure high video quality and a direct visualization on a single erythrocyte scale. pCLE recordings were carried out for 3 min resulting in a real-time imaging of more than 2000 frames. Using the videomosaicing function provided by the analysis software which enables the input frames to be aligned, we also obtained the reconstruction of the scanned panoramas of the mucosa. Through this technique we assessed: 1) the mucosal architecture; 2) the vascular density and vessel morphology; 3) the efficiency of the blood flow. Endoscopists provided an initial evaluation of the various angiogenic parameters in real time (on-line evaluation). The images were digitally stored and reviewed by a single investigator who is fully trained and proficient in confocal image interpretation with the dedicated software package (Cellvizio Viewer, Mauna Kea Technologies), which also enables image correction and stabilization. This investigator was blinded to any clinical, endoscopical, or histopathological information (off-line evaluation). The evaluation of the extent and quality of the intra-tumoral angiogenesis was determined based on the presence of tortuous and large sized vessels, the vessels’ leakage and the presence of defective flow. Surgical specimens were submitted for pathological examination. Sections were obtained from tumor lesions and analyzed by immunohistochemistry using an antibody specific for the endothelial marker CD31 (Clone JC70A, DakoCytomation, Italy) to visualize the vessels.

### Statistical analysis

Fisher or McNemar tests were carried out to assess differences between frequencies, as appropriate. All tests were two-tailed and results were considered statistically significant for a p-value < 0.05.

## Electronic supplementary material

Supplemental information

Video S1
