# Supplementary material for: Probe-based confocal laser endomicroscopy for in vivo evaluation of the tumor vasculature in gastric and rectal carcinomas
Source: Sci Rep. 2017 Aug 29;7:9819. doi: 10.1038/s41598-017-10963-1 (PMC5575283; doi:10.1038/s41598-017-10963-1)
Supplement: Supplementary file 1 — Supplemental information [file 41598_2017_10963_MOESM1_ESM.pdf]

Supplementary information

Probe-based confocal laser endomicroscopy for in vivo evaluation of the tumor vasculature in gastric and rectal carcinomas.

Paola Spessotto, Mara Fornasarig, Eliana Pivetta, Stefania Maiero, Raffaella Magris, Maurizio Mongiat, Vincenzo Canzonieri, Paolo De Paoli, Antonino De Paoli, Angela Buonadonna, Diego Serraino, Chiara Panato, Claudio Belluco, Renato Cannizzaro

Table S1. Clinic-pathological characteristics of patients.

**GASTRIC**			**RECTAL**		
	**N**	**%**		**N**	**%**
All cases	35	100	All cases	91	100
Revaluated post CRT	11	31	Revaluated post CRT	47	52
Male	21	60	Male	65	71.4
Mean Age	59(48-71)		Mean Age	64(39-83)	
Female	14	40	Female	26	28.6
Mean Age	60(44-68)		Mean age	64(37-78)	
**Histologic type**			**Pre CRT- clinical stage**		
Diffuse	15	42.8	T2/T3N0	38	41.8
Intestinal	20	57.2	TxN+	37	40.7
**Tumor site**					
Fundus	6	17.1			
Corpus	23	65.7			
Antrum	6	17.2			
**Pre CRT- clinical stage**					
T2/T3N0	14	40			
TxN+	21	60			

CRT = chemio/radio therapy

Video S1. Representative sequences of defective flow in a rectal cancer patient with an angiogenic score 4.

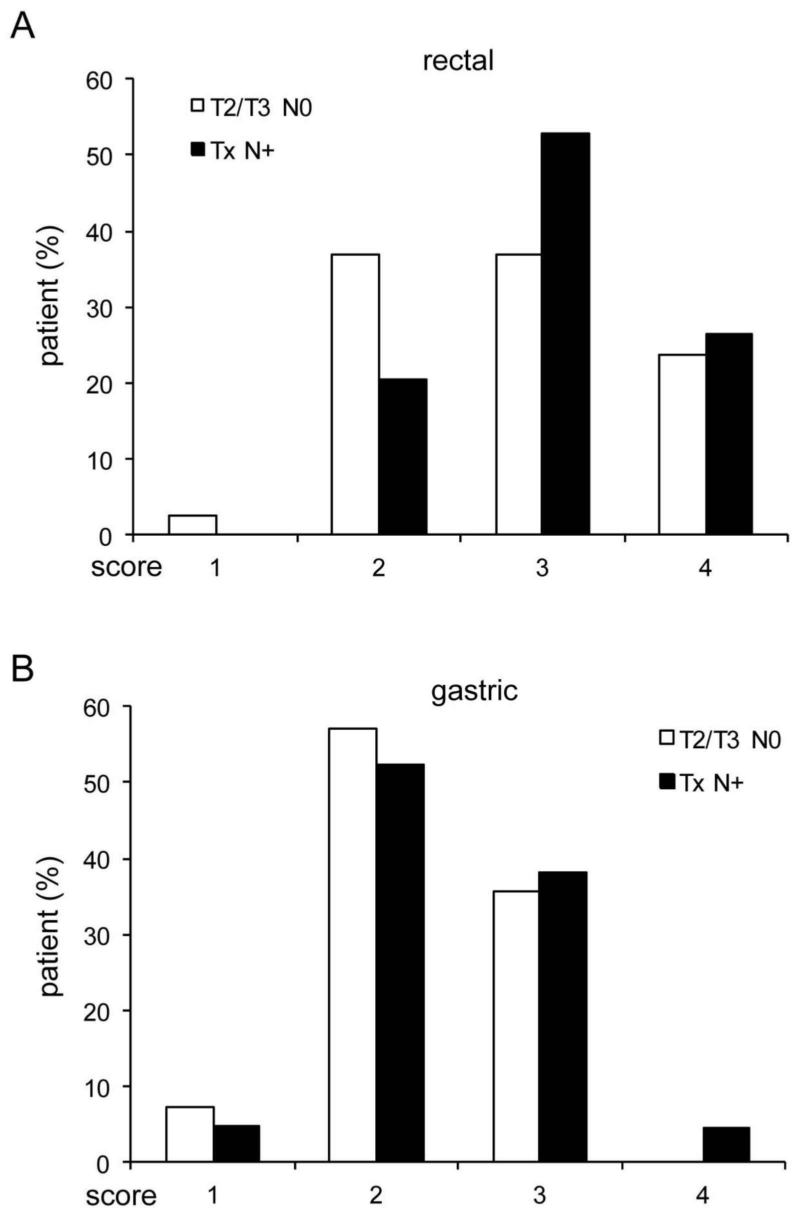

Fig. S1. Graphs showing the association between angiogenic score and tumor staging in rectal (A) and gastric (B). Tumors were divided in two groups (T2/T3N0 and TxN+) according to the presence of lymph node infiltration as assessed by ultrasonography during standard endoscopy.

Table S2. Percentage of gastric cancer patients displaying the morphological and functional parameters taken into account to generate the angiogenic score assessed before and after therapy.

	Gastric (N=11)		
	First pCLE N (%)	Second pCLE N (%)	McNemar
**Leakage**			
Yes	10 (90.9)	11 (100.0)	
No	1 (9.1)	0 (0.0)	
**Tortuous vessels**			
Yes	9 (81.8)	5 (45.5)	*p-value=0.10*
No	2 (18.2)	6 (54.5)	
**Large vessels**			
Yes	9 (81.8)	8 (72.7)	*p-value=0.65*
No	2 (18.2)	3 (27.3)	
**Defective flow**			
Yes	0 (0.0)	0 (0.0)	
No	11 (100.0)	11 (100.0)	
